# Network Pharmacology as a Tool to Explore the Therapeutic Mechanism of *Opuntia Ficus-Indica* (Nopal) in Type 2 Diabetes and Colorectal Cancer

**DOI:** 10.2174/0115680266398981251117100048

**Published:** 2026-01-09

**Authors:** Juan Manuel Guzmán-Flores, Fernando Martínez-Esquivias, Julián Paul Martínez-Galán, Sandra Lucia Restrepo-Mesa, Mario Alberto Isiordia-Espinoza, Mabel R Challapa-Mamani, Orfanel Sebastián Pineda-Arzate

**Affiliations:** 1 Departamento de Ciencias de la Salud, Centro Universitario de los Altos, Universidad de Guadalajara, Tepatitlán de Morelos, Jalisco, México;; 2 Departamento de Ciencias Pecuarias y Agrícolas, Centro Universitario de Los Altos, Universidad de Guadalajara, Tepatitlán de Morelos, Jalisco México;; 3 Laboratorio Alimentación y Nutrición Humana, Escuela de Nutrición y Dietética, Universidad de Antioquia, Medellín, Antioquia, Colombia;; 4 Grupo de Investigación Alimentación y Nutrición Humana, Escuela de Nutrición y Dietética, Universidad de Antioquia, Medellín, Antioquia, Colombia;; 5 Instituto de Investigación en Ciencias Médicas, Departamento de Clínicas, División de Ciencias Biomédicas, Centro Universitario de los Altos, Universidad de Guadalajara, Tepatitlán de Morelos, Jalisco, México;; 6 Escuela de Medicina, Universidad Cesar Vallejo, Trujillo, Perú;; 7 Facultad de Medicina, Benemérita Universidad Autónoma de Puebla, Puebla, México

**Keywords:** Nopal, Cancer, Colorectal, Diabetes, Network pharmacology, Bioinformatics

## Abstract

**Introduction:**

Considering the shared physiological mechanisms between type 2 diabetes (T2D) and colorectal cancer (CRC), it is plausible that certain compounds may exert therapeutic effects on both diseases. Opuntia ficus-indica (nopal) has been traditionally used to manage these conditions. This study aims to elucidate the molecular mechanisms through which nopal exerts its effects on T2D and CRC.

**Methods:**

Bioactive compounds of nopal, their molecular targets, and genes associated with T2D and CRC were identified from public databases. Gene Ontology (GO) analysis, metabolic pathway analysis, protein-protein interaction (PPI) network construction, and molecular docking were conducted to investigate the shared molecular targets.

**Results:**

Nopal contains bioactive compounds that interact with molecular targets common to both T2D and CRC. These shared targets are implicated in lipid metabolism, apoptosis, kinase activity, interleukin-related pathways (IL-2 and IL-3), inflammation, gastrin signaling, and other critical processes. Key molecular targets identified include HSP90AA1 and MAPK8, while the principal bioactive compounds of nopal are eriodictyol and aromadendrin.

**Discussion:**

The identification of eriodictyol and aromadendrin as modulators of HSP90AA1 and MAPK8 elucidates a pleiotropic mechanism underlying the link between type 2 diabetes and colorectal cancer. By modulating apoptotic and inflammatory pathways, these bioactive compounds offer a promising foundation for developing dual-action therapies targeting both metabolic and oncogenic pathways in patients with comorbid conditions.

**Conclusion:**

The bioactive compounds of nopal engage multiple biological pathways relevant to T2D and CRC, suggesting that this plant may serve as a promising pharmacological candidate for the management of these diseases.

## 1 INTRODUCTION

Type 2 diabetes (T2D) is a complex, multifactorial disease that affects multiple organs and disrupts carbohydrate, lipid, and protein metabolism. It is characterized by elevated blood glucose levels [1] and has been increasing in prevalence worldwide, particularly in developing countries such as Mexico, which ranks among the top 10 most affected nations [2, 3]. The etiology of T2D involves insulin resistance, impaired insulin secretion, and alterations in the immune, gastrointestinal, and nervous systems [1, 4].

T2D has also been linked to a wide range of other diseases, including cancer. Colorectal cancer (CRC), as defined by the American Cancer Society, originates in the colon or rectum and is characterized by abnormal cell growth that forms clumps along the inner lining of these organs [5]. Globally, CRC ranked third in incidence and second in mortality in 2022. In Mexico, it is the most common type of cancer by incidence [6, 7].

Several studies have reported an association between T2D and CRC, with proposed pathophysiological mechanisms including hyperinsulinemia, activation of the insulin-like growth factor (IGF) axis, hyperglycemia, inflammation induced by adipose tissue dysfunction, gastrointestinal motility disorders, and impaired immune surveillance [8, 9]. These overlapping mechanisms suggest that some therapeutic agents may have efficacy against both T2D and CRC. Emerging strategies for managing these diseases include nanoparticles and plant-derived compounds such as curcumin [10-13].

One promising therapeutic option is Opuntia ficus-indica, commonly known as Nopal. This plant is native to arid and semi-arid regions and is distributed across Mexico, Latin America, South Africa, and Mediterranean countries. Nopal is widely used in health, nutrition, and cosmetics, with products including tea, jam, juice, and seed oil. Traditionally, it has been employed as a remedy for various conditions, including metabolic syndrome, type 2 diabetes, obesity, non-alcoholic fatty liver disease, rheumatism, cerebral ischemia, cancers, and viral and bacterial infections [14].

Several studies have reported beneficial effects of Nopal on T2D, demonstrating reductions in serum glucose levels, and on CRC, showing antiproliferative and proapoptotic effects in cancer cell lines [15, 16]. However, the molecular mechanisms underlying these effects, particularly the roles of the bioactive compounds present in Nopal, remain poorly understood.

Therefore, this study aims to investigate the bioactive components, molecular targets, and metabolic pathways through which Nopal exerts its effects on T2D and CRC, using network pharmacology and molecular docking approaches.

## 2 MATERIALS AND METHODS

### 2.1 Screening of Possible Bioactive Compounds from Opuntia Ficus-indica

To identify the bioactive compounds of Opuntia ficus-indica (Nopal), we used the IMPPAT database (Indian Medicinal Plants, Phytochemistry, and Therapeutics) [17]. The search was performed using the plant’s scientific name. From the resulting compounds, we applied two selection criteria: a bioavailability score greater than 0.30 and a weighted quantitative estimate of drug-likeness (QEDw) score greater than 0.30 [18, 19].

### 2.2 Prediction of Targets for Each Compound of Opuntia Ficus-indica

The SwissTargetPrediction database was used to identify the potential molecular targets of the Nopal bioactive compounds. Canonical SMILES structures were uploaded, and targets were predicted under the criteria “Homo sapiens” and a probability ≥0.1 [20]. In addition, the PharmMapper database was used to perform pharmacophore-based reverse screening. The mol2 files of the selected bioactive compounds were uploaded, and target prediction was carried out using the settings “Homo sapiens” and a normalized fit score ≥0.5 [21].

### 2.3 Collection of Target Genes for Type 2 Diabetes and Colorectal Cancer

The target genes associated with both diseases were collected from the Comparative Toxicogenomics Database (CTD) [22], MalaCards [23], and DisGeNET [24]. In CTD, the keywords “Diabetes Mellitus, Type 2” and “Colorectal Neoplasms” were used, selecting the “curated” option under Gene-Disease Association and specifying the organism as Homo sapiens. In MalaCards, the terms “Type 2 Diabetes Mellitus (T2D)” and “Colorectal Cancer (CRC)” were queried. For DisGeNET, we searched using the keywords “Diabetes Mellitus, Non-Insulin-Dependent” and “Colorectal Carcinoma, C0009402,” and filtered gene-disease associations using a GDA score > 0.1.

The intersecting genes shared by T2D, CRC, and the predicted targets of Nopal bioactive compounds were identified using a Venn diagram generated with the Venny 2.1 platform.

### 2.4 Analyzing Gene Ontology (GO) and Kyoto Encyclopedia of Genes and Genomes (KEGG) Pathway Enrichment

Genes common to Nopal bioactive compound targets and those associated with T2D and CRC were analyzed using the ShinyGO 0.80 platform [25], a graphical and intuitive web-based tool that facilitates the extraction of functional insights from gene sets. This analysis aimed to identify the biological functions in which Nopal bioactive compounds may participate across both diseases. Gene enrichment analyses were conducted for Biological Process, Molecular Function, and Cellular Component categories. Additionally, metabolic pathway enrichment was performed using the ToppGene Suite, specifically the ToppFun module [26]. The False Discovery Rate (FDR) for p-value adjustment was controlled using the Benjamini-Hochberg method.

### 2.5 Construction of the Nopal Bioactive Compounds-targets-metabolic Pathways-diseases Network

An interaction network between these parameters was created using the Cytoscape platform to visually represent the relationship between Nopal's bioactive compounds, their targets, the metabolic pathways in which these targets participate, and the two diseases studied [27].

### 2.6 Molecular Docking

For the molecular docking analysis, the structures of the Nopal bioactive compounds were first obtained in 3D mol2 format from the IMPPAT platform [17]. Protein structures were retrieved from the AlphaFold Protein Structure Database [28], selecting only reviewed entries corresponding to Homo sapiens. These ligand and protein files were imported into PyRx, where docking was performed using the Vina Wizard module with an exhaustiveness parameter of 8 and the maximum search box configuration [29]. From the resulting docking poses, the model exhibiting the lowest binding affinity energy was selected. The selected complexes were subsequently downloaded and visualized in two-dimensional conformation using BIOVIA Discovery Studio.

## 3 RESULTS

### 3.1 Bioactive Compounds and Targets from Opuntia Ficus-indica

Through the IMPPAT platform, we found 17 compounds in the Nopal plant. However, after applying the filters of an OB of ≥0.30 and QEDw of ≥0.30, we were left with 13 bioactive compounds from the Nopal plant. Table **1** shows the 13 bioactive compounds and some of their characteristics. Subsequently, their targets were inferred from these 13 bioactive compounds using the PharmMapper and SwissTarget Prediction platforms. A total of 430 molecules were identified as potentially interacting with the 13 bioactive compounds present in nopal.

### 3.2 Genes Related to Type 2 Diabetes and Colorectal Cancer

Using MalaCards, DisGeNet, and Comparative Toxicogenomics, we identified 923 T2D-related genes and 1,896 CRC-related genes using omics databases. Subsequently, we performed a Venn diagram to identify the common genes between these two diseases and the targets of Nopal bioactive compounds. As shown in Fig. (**1**), T2D and CRC share 334 genes in common; Nopal bioactive compounds affect more CRC (153) than T2D (108), and 55 genes are shared between T2D, CRC, and Nopal targets.

### 3.3 Gene Ontology Enrichment and Metabolic Pathways

Gene Ontology enrichment analysis identified significantly enriched terms (p < 0.001) in genes modulated by nopal compounds in colorectal cancer and type 2 diabetes. The most enriched biological processes included “responses to lipid,” “programmed cell deaths,” and “apoptotic processes.” The terms “cellular responses to chemical stimuli” and “responses to organic substances” suggest that nopal compounds activate complex cellular signaling cascades. The cellular components exhibited substantial enrichment in “caveolae,” “plasma membrane rafts,” and “focal adhesion,” indicating the modulation of specialized membrane domains and cell adhesions. The presence of terms such as “membrane microdomains” and “vesicle lumen” suggests that the effects extend to multiple subcellular compartments, including mitochondria and the extracellular space. The molecular functions that exhibited enrichment included “nuclear receptor activities,” “protein kinase activity,” and “small molecule binding,” with gene counts ranging from 10 to 40 genes per term. The terms “transferase activities transferring phosphorus-containing groups” and “protein serine/threonine/tyrosine kinase activities” confirm the modulation of phosphorylation cascades, which are fundamental mechanisms in cell signaling. The distribution of enrichment values (10-50) and associated gene counts (5-40) demonstrates that nopal compounds modulate extensive molecular networks encompassing multiple aspects of cellular function, providing solid molecular evidence for their pleiotropic effects in both pathologies through modulation of critical pathways of cell survival, programmed death, and metabolic signaling. The top 10 Gene Ontology enrichments are shown in Fig. (**2**).

Table **2** presents the top 10 enriched metabolic pathways, highlighting those related to cancer, cytokine signaling, inflammation, and signaling pathways involving estrogen, prolactin, and progesterone. To clarify the relationship between the bioactive compounds in nopal, their corresponding targets, and the metabolic pathways influenced in both diseases, an interaction network was constructed. As shown in Fig. (**3**), a total of 36 target proteins were associated with 13 bioactive nopal compounds across eight primary signaling pathways. The network exhibited a tripartite structure, linking compounds such as eriodictyol, quercetin, kaempferol, and β-sitosterol with key proteins implicated in type 2 diabetes and colorectal cancer. Among these, AKT1, SRC, MAPK14, MAPK8, and HSP90AA1 emerged as hub nodes with the highest degree of connectivity, serving as central regulatory points in the PI3K-Akt, MAPK, and interleukin signaling pathways. Given their extensive interactions, subsequent analyses will focus on these five hub proteins. This molecular convergence between T2D and CRC supports the presence of shared therapeutic mechanisms.

### 3.4 Docking Molecular

Molecular docking is a computational method used to predict the most favorable conformation of a ligand when bound to a target protein, yielding an estimated binding affinity expressed as ΔG (kcal/mol). Typically, ΔG values below −5 kcal/mol indicate good interactions, and values below −7 kcal/mol reflect strong interactions. Based on the key targets identified in Fig. (**3**), we conducted molecular docking between the five proteins with the highest number of interactions and the thirteen bioactive compounds in nopal. The results are summarized in Table **3**. Notably, the interactions of HSP90AA1 with aromadendrin and eriodictyol, as well as MAPK8 with β-sitosterol, all exhibited ΔG values lower than −9 kcal/mol, indicating highly favorable binding. To further characterize these interactions, we analyzed the specific bonds involved, which are depicted in Fig. (**4**).

Additionally, the ΔG values demonstrated stronger affinities for several nopal metabolites compared with known inhibitors of HSP90AA1 and MAPK8. In experimental data, 17-AAG showed a binding affinity of −6.9 kcal/mol with HSP90AA1, while SP600125 exhibited a ΔG of −8.3 kcal/mol with MAPK8. In contrast, eriodictyol (−9.3 kcal/mol) and aromadendrin (−9.2 kcal/mol) displayed more favorable binding to HSP90AA1, and β-sitosterol (−9.4 kcal/mol) surpassed SP600125 in affinity for MAPK8. These results identify the three compounds as promising candidates for future experimental validation.

The docking analysis revealed three major classes of non-covalent interactions contributing to the stability of protein-ligand complexes. Hydrogen bonds provide binding specificity through directional interactions between donor and acceptor groups. Alkyl and π-alkyl interactions arise from hydrophobic contacts between ligand aliphatic groups and hydrophobic protein residues, stabilizing the complex *via* the hydrophobic effect. π-π and π-σ stacking interactions occur between ligand aromatic rings and aromatic protein residues such as PHE, TYR, and TRP. The specific interacting residues are detailed in Fig. (**4A**-**C**). In the HSP90AA1-aromadendrin complex, a hydrogen bond is formed with ASP93, accompanied by π-π stacking with PHE138 and TRP162. In the HSP90AA1-eriodictyol complex, alkyl interactions occur with VAL40, LEU168, LYS55, and ILE32. For the MAPK8-β-sitosterol complex, two hydrogen bonds form with residues TYR139 and ASN51. Collectively, these diverse interactions support the formation of stable protein-ligand complexes and reinforce the therapeutic potential of nopal bioactive compounds in modulating pathways relevant to colorectal cancer and type 2 diabetes.

## 4 DISCUSSION

Type 2 diabetes and colorectal cancer are multifactorial diseases with high prevalence in all world regions. Despite the wide array of drugs available for their treatment, the incidence of these diseases continues to increase. Moreover, conventional drugs often present adverse effects, highlighting the need for alternative therapies, such as plant-derived compounds like Nopal. In this study, we explored the molecular mechanisms through which Nopal bioactive compounds may exert effects on T2D and CRC.

We identified 13 bioactive compounds in Nopal, targeting 430 molecular sites in humans. Additionally, 923 genes associated with T2D and 1896 genes associated with CRC were identified, with 55 genes common to both diseases. Gene ontology enrichment and metabolic pathway analyses revealed that these shared genes are involved in lipid regulation, MAP kinase activity, insulin receptor binding, and metabolic pathways related to cytokines, estrogen, and inflammation. Among these genes, five, AKT1, SRC, MAPK14, MAPK8, and HSP90AA1, stood out due to their high level of interaction with Nopal bioactive compounds, a finding further supported by molecular docking.

All 13 selected Nopal compounds exhibited similar oral bioavailability (OB), approximately 0.55. However, two compounds, eriodictyol and aromadendrin, displayed higher weighted quantitative estimates of drug-likeness (QEDw), 0.60 and 0.63, respectively. Eriodictyol is a flavonoid found in medicinal plants, citrus fruits, and vegetables, with reported antioxidant, anti-inflammatory, anticancer, neuroprotective, cardioprotective, antidiabetic, anti-obesity, and hepatoprotective properties [30]. Aromadendrin, a flavonoid present in a wide variety of plants, promotes insulin-mediated glucose uptake *via* the Akt pathway and exhibits cytotoxic and anti-inflammatory activity [31]. Therefore, both eriodictyol and aromadendrin possess anti-inflammatory and pharmacological properties that could be beneficial in treating T2D and CRC.

Our analysis revealed a greater number of genes associated with CRC than T2D. Notably, 334 genes were common to both diseases, emphasizing their close relationship, which is consistent with previous reports [8, 9]. The intersection of genes related to CRC, T2D, and Nopal targets yielded 55 common genes. There were more shared genes between CRC and Nopal targets (153) than between T2D and Nopal targets (108), suggesting that Nopal compounds may exert a stronger effect on CRC than on T2D.

Gene ontology enrichment analysis revealed that Nopal bioactive compounds are involved in biological processes related to lipids. Altered lipid metabolism is evident in T2D, and changes in lipid composition and metabolism have also been reported in CRC [32]. Studies have shown that Opuntia ficus-indica decreases serum lipid levels [33], supporting the potential of Nopal to regulate lipid metabolism in both diseases. Apoptosis was another significantly enriched biological process. Programmed cell death is a key mechanism in the deterioration of pancreatic beta cells in diabetes, influenced by inflammation, oxidative stress, and endoplasmic reticulum stress [34]. Similarly, colorectal cancer cells often activate survival pathways to evade apoptosis, involving molecules such as Bcl-2, Bax, and caspases [35]. Interestingly, Nopal compounds have been reported to modulate apoptosis in both CRC and T2D [36, 37], further supporting their therapeutic potential.

One of the enriched cellular components identified was the mitochondrion. This organelle is affected by insulin resistance, a hallmark of T2D, and is also altered in CRC. Interestingly, Nopal has been reported to regulate mitochondrial function [36, 38], suggesting that it may modulate this organelle in both T2D and CRC.

Regarding molecular functions, nuclear receptor activity was enriched. Nuclear receptor ligands are often lipid-based, such as steroid hormones, and their binding typically occurs in the cytosol, followed by translocation to the nucleus. Estrogen receptor beta (ESR2) is one such molecule; it has been proposed as a therapeutic target for preventing CRC and, in diabetes, regulates the translocation of glucose transporter 4 (GLUT4) [39, 40]. Since ESR2 is a target of Nopal bioactive compounds, these compounds may modulate ESR2 activity, impacting both T2D and CRC.

Enriched metabolic pathways included signaling by interleukins, particularly IL-2 and IL-3. IL-2 plays a central role in T cell-mediated immune responses and is elevated in CRC patients; it is also altered in T2D [41, 42]. Similar alterations in IL-3 levels have been reported in both CRC and T2D [43, 44]. Although the direct effect of Nopal compounds on IL-2 and IL-3 has not been reported, this plant has been shown to regulate other interleukins, suggesting a likely regulatory effect on IL-2 and IL-3 [45, 46]. The gastrin signaling pathway was also enriched. Gastrin promotes the proliferation of colonic epithelial cells and has been proposed as a therapeutic target in CRC [47]. In T2D, gastrin can enhance pancreatic beta-cell neogenesis when administered with incretins [48]. Therefore, Nopal compounds may exert beneficial effects through the modulation of gastrin signaling.

From the interaction network analysis, five critical proteins were identified: AKT1, SRC, MAPK14, MAPK8, and HSP90AA1. Molecular docking assays revealed three notable interactions: HSP90AA1-Aromadendrin, HSP90AA1-Eriodictyol, and MAPK8-beta sitosterol. HSP90AA1 participates in a dissociation process that disrupts normal protein folding, promoting ubiquitination and degradation of oncogenic proteins *via* E3 ligases such as CHIP and FBXL6 [49, 50]. In CRC, HSP90AA1 inhibition destabilizes key oncoproteins, triggering proteasomal degradation [49]. MAPK8 regulates epithelial-mesenchymal transition (EMT) by phosphorylating Smad3 and activating β-catenin, promoting invasion and metastasis [51]. Its modulation inhibits pathological EMT in CRC and improves insulin sensitivity in T2D [52]. Eriodictyol and Aromadendrin’s dual affinity for these targets suggests potential synergistic therapeutic effects for both diseases.

Eriodictyol and Aromadendrin also exhibited the highest QEDw, reinforcing their potential as pharmacological agents. Both compounds possess anti-inflammatory properties, which are advantageous for treating CRC and T2D [30, 31].

Heat shock protein HSP90AA1 is a chaperone involved in multiple biological functions and has been proposed as a therapeutic target in T2D based on its high connectivity with other proteins and its ability to bind plant-derived bioactive compounds [53-55]. Similar findings have been reported in CRC, with HSP90AA1 identified as a potential therapeutic target through bioinformatics, *in vitro*, and *in vivo* studies [56, 57]. These results support the notion that Eriodictyol and Aromadendrin can inhibit HSP90AA1, reinforcing their potential as bioactive therapeutic agents derived from Nopal.

MAPK8 belongs to the MAP kinase family, which serves as an integration point for multiple biochemical signals and is involved in diverse cellular processes, including cell proliferation, differentiation, transcription regulation, and development. MAPK8 has been previously identified as a therapeutic target for both T2D and CRC using network pharmacology approaches, similar to our study [58, 59]. Therefore, MAPK8 may represent a therapeutic target for β-sitosterol, a bioactive compound from Nopal, potentially exerting beneficial effects in both T2D and CRC.

## STUDY LIMITATIONS

Acknowledging the limitations of in silico studies, future experimental research should include targeted knockdown or overexpression of HSP90AA1 and MAPK8 using CRISPR-Cas9 technology in colorectal and pancreatic cancer cell lines. These experiments should incorporate functional assays to assess cell viability, apoptosis, and cell cycle progression, along with *in vivo* validation in murine models of T2D and CRC.

Our findings also highlight eriodictyol and aromadendrin as key bioactive compounds with promising pharmacological properties. Molecular docking revealed strong interactions for HSP90AA1-aromadendrin, HSP90AA1-eriodictyol, and MAPK8-β-sitosterol. Notably, 55 genes were identified as common targets of T2D, CRC, and Nopal bioactive compounds, enriched in key signaling pathways, including PI3K-Akt, MAPK, and interleukin pathways. These results provide a robust foundation for developing dual therapeutic strategies, particularly given Nopal’s established safety as a traditional food, which supports its potential clinical application.

## CONCLUSION

In conclusion, Opuntia ficus-indica (Nopal) contains bioactive compounds that modulate molecular pathways shared by both type 2 diabetes and colorectal cancer. These common targets are involved in lipid regulation, apoptosis, kinase activity, and metabolic pathways associated with interleukins (IL-2 and IL-3), inflammation, gastrin signaling, and other critical processes. Among the key molecular targets identified are HSP90AA1 and MAPK8, while the principal bioactive compounds of Nopal are eriodictyol and aromadendrin. These findings indicate that Nopal bioactive compounds participate in multiple biological functions relevant to type 2 diabetes and colorectal cancer, highlighting this plant as a promising pharmacological option for the management of these diseases.

## AUTHORS’ CONTRIBUTIONS

The authors confirm their contribution to the paper as follows: Conceptualization, JM G-F; methodology, JM G-F, F M-E, JP M-G, S L R-M, MA I-E, MR C-M, OS P-A; formal analysis, JM G-F, F M-E, JP M-G, S L R-M, MA I-E, MR C-M, OS P-A; writing—original draft preparation, JM G-F; writing—review and editing, JM G-F, F M-E, JP M-G, S L R-M, MA I-E, MR C-M, OS P-A; All authors have read and agreed to the published version of the manuscript.

## Figures and Tables

**Fig. (1):**
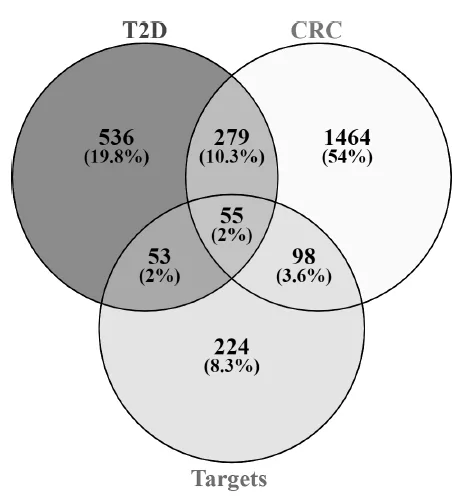
Venn diagram of Nopal targets related to type 2 diabetes and colorectal cancer.

**Fig. (2):**
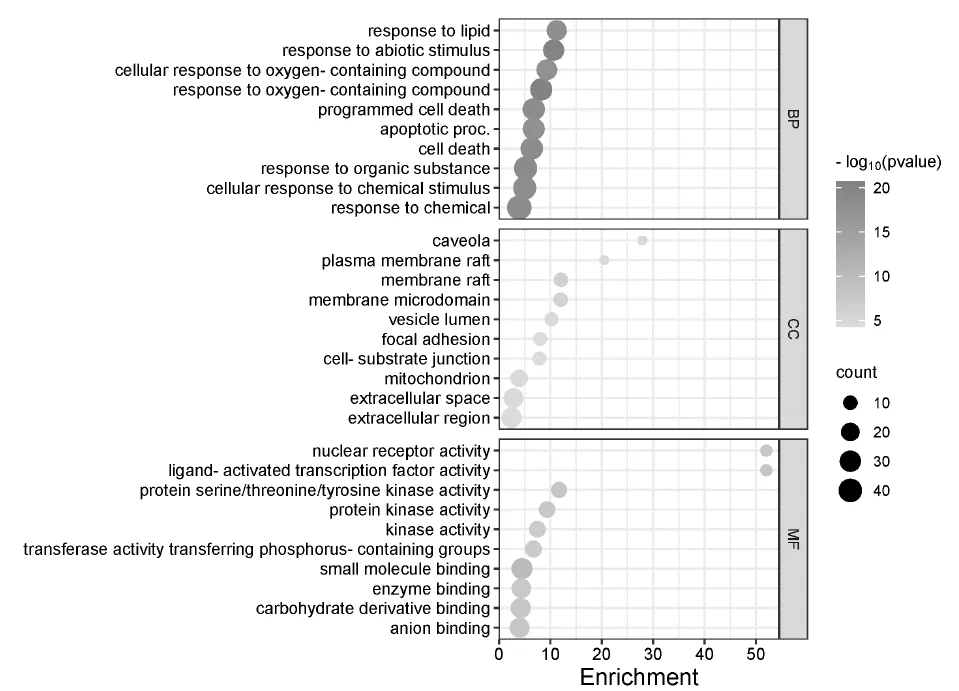
Gene ontology enrichment. Biological Process, BP: Cellular Component, CC; and Molecular Function, MF.

**Fig. (3):**
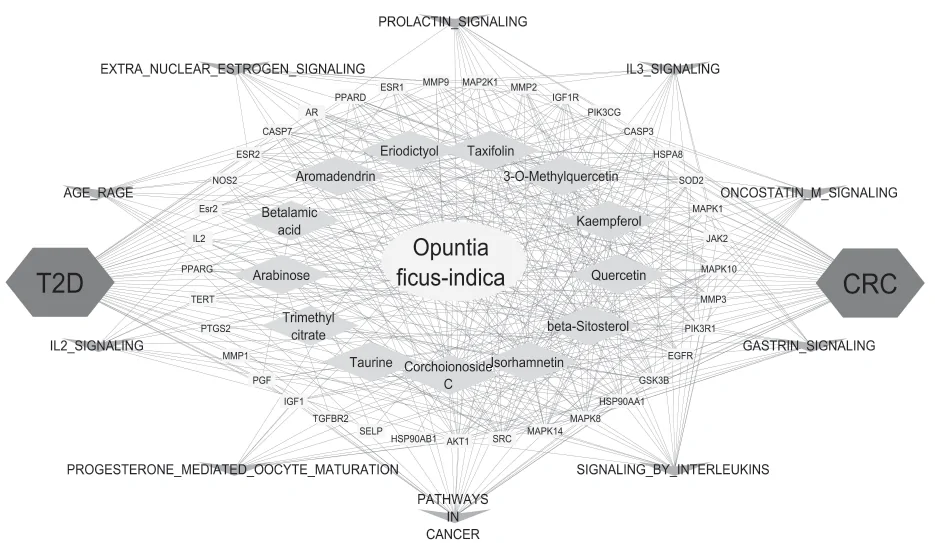
Nopal bioactive compounds-targets-metabolic pathways-diseases network.

**Fig. (4):**
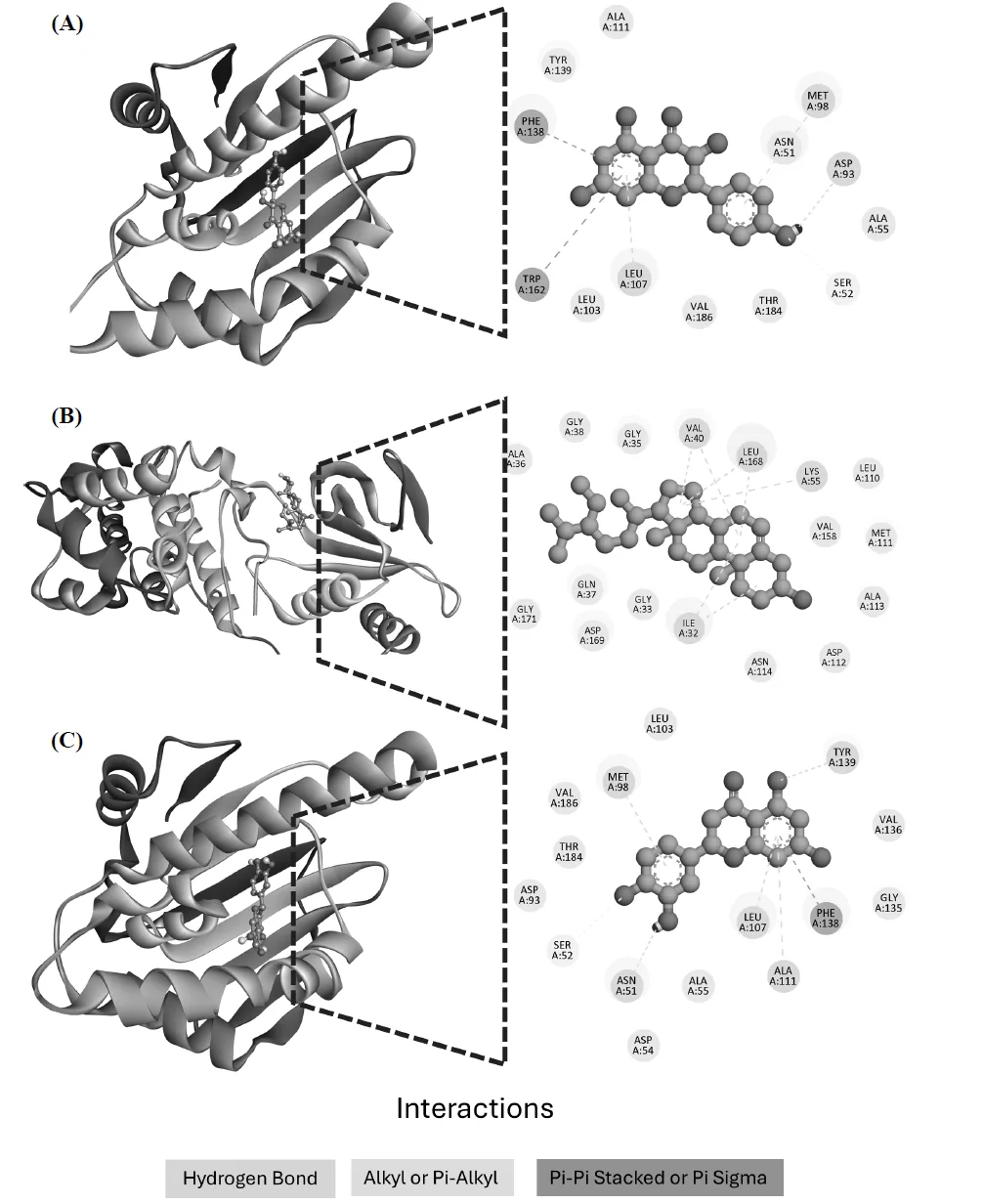
Top 3 molecular docking between Nopal bioactive compounds and their targets in T2D and CRC. **A**). HSP90AA1-Aromadendrin. **B**) HSP90AA1-Eridictyol. **C**) MAPK8-beta sitosterol.

**Table 1:** Bioactive compounds of Opuntia ficus-indica and their MW, OB, and QEDw.

**Molecule ID**	**Molecule Name**	**MW**	**OB**	**QEDw**	**PubChem CID**
IMPHY014836	Beta-Sitosterol	414.72	0.55	0.44	222284
IMPHY011967	Taxifolin	304.25	0.55	0.50	439533
IMPHY011540	3-O-Methylquercetin	316.27	0.55	0.54	5280681
IMPHY008724	Isorhamnetin	316.27	0.55	0.57	5281654
IMPHY007042	Trimethyl citrate	234.2	0.55	0.48	74112
IMPHY004676	Betalamic acid	211.17	0.56	0.43	5281176
IMPHY004619	Quercetin	302.24	0.55	0.43	5280343
IMPHY004388	Kaempferol	286.24	0.55	0.55	5280863
IMPHY004219	Corchoionoside C	386.44	0.55	0.40	10317980
IMPHY004187	Arabinose	150.13	0.55	0.33	5460291
IMPHY004038	Eriodictyol	288.26	0.55	0.60	440735
IMPHY002073	Aromadendrin	288.26	0.55	0.63	122850
IMPHY000040	Taurine	125.15	0.55	0.46	1123

**Table 2:** Top 10 enriched metabolic pathways affected by Nopal on T2D and CRC.

**Metabolic Pathway Name**	**ID**	**Source**	**p-value**	**Hit in Query List**
KEGG_PATHWAYS_IN_CANCER	M12868	KEGG Pathways	3.11E-20	TGFBR2, IGF1, IGF1R, EGFR, PIK3CG, PIK3R1, CASP3, AKT1, MMP1, MMP2, MAPK1, PPARD,P PARG, MMP9, MAPK8, MAPK10, MAP2K1, NOS2, PGF, PTGS2, AR, GSK3B, HSP90AA1
WP_GASTRIN_SIGNALING_PATHWAY	M39866	WikiPathways	9.57E-15	JAK2, MAPK14, EGFR, PIK3R1, SRC, CASP3, AKT1, MAPK1, PPARG, MAPK8, MAP2K1, IL2,PTGS2, GSK3B
WP_ONCOSTATIN_M_SIGNALING_PATHWAY	M39562	WikiPathways	9.57E-15	JAK2, MAPK14, PIK3R1, SRC, CASP3, CASP7, AKT1, MMP1, MAPK1, MMP3, MAPK8, MAP2K1
WP_AGERAGE_PATHWAY	M39420	WikiPathways	9.57E-15	JAK2, MAPK14, EGFR, SRC, CASP3, AKT1,MMP2, MAPK1, MMP9, MAPK8, MAP2K1, NOS2
REACTOME_EXTRA_NUCLEAR_ESTROGEN_SIGNALING	M27874	Reactome Pathways	3.91E-14	IGF1R, EGFR, PIK3R1, ESR1, ESR2, SRC, AKT1, MMP2, MAPK1, MMP3, MMP9, HSP90AA1
WP_PROLACTIN_SIGNALING_PATHWAY	M39601	WikiPathways	3.91E-14	JAK2, MAPK14, PIK3CG, PIK3R1, SRC, CASP3, AKT1, MAPK1, MAPK8, MAP2K1, NOS2, GSK3B
WP_IL3_SIGNALING_PATHWAY	MM15879	WikiPathways	9.25E-13	SELP, JAK2, MAPK14, PIK3R1, SRC, AKT1, MMP2, MAPK1, MMP9, MAPK8, MAP2K1, GSK3B
REACTOME_SIGNALING_BY_INTERLEUKINS	M874	Reactome Pathways	2.13E-12	JAK2, MAPK14, PIK3R1, CASP3, AKT1, MMP1, MMP2, MAPK1, MMP3, MMP9, MAPK8, MAPK10, MAP2K1, IL2, NOS2, PTGS2, HSPA8, HSP90AA1, SOD2
KEGG_PROGESTERONE_MEDIATED_OOCYTE_MATURATION	M3578	KEGG Pathways	4.91E-12	IGF1, IGF1R, MAPK14, PIK3CG, PIK3R1, AKT1, MAPK1, MAPK8, MAPK10, MAP2K1, HSP90AA1
WP_IL2_SIGNALING_PATHWAY	MM15915	WikiPathways	2.31E-14	JAK2, MAPK14, PIK3CG, PIK3R1, AKT1, MAPK1, MAPK8, MAP2K1, IL2, TERT, HSP90AA1

**Table 3:** Binding energetics (kcal/mol) obtained from molecular docking between Nopal bioactive compounds and the top 5 targets of T2D and CRC.

**Bioactive Compound**	**AKT1**	**SRC**	**MAPK14**	**MAPK8**	**HSP90AA1**
Eriodictyol	-8.3	-8.6	-8.1	-8.5	-9.3*
Aromadendrin	-8	-8.6	-8	-8.3	-9.2*
Quercetin	-8.3	-8.8	-8.3	-8.4	-7.9
Taxifolin	-8.4	-8.9	-8	-8.2	-7.6
Beta-Sitosterol	-7.8	-8.4	-8.8	-9.4*	-6.2
Isorhamnetin	-7.9	-8.5	-8.2	-8.6	-7.3
Kaempferol	-8	-8.4	-8.3	-8.4	-7.2
3-Methoxyluteolin	-8	-8	-8	-8.1	-6.8
Corchoionoside C	-7.3	-7.9	-8.2	-8	-6.8
Betalamic acid	-6.2	-6.3	-6.5	-6.2	-6.1
Trimethyl citrate	-5	-4.9	-4.9	-4.6	-4.4
Arabinose	-4.6	-5	-4.3	-4.4	-4.9
Taurine	-3.7	-4.2	-3.8	-3.8	-4.5

**Note:** *Interactions with lower binding energetics (kcal/mol).

## Data Availability

The data supporting this study's findings are available from the corresponding author upon reasonable request.

## LIST OF ABBREVIATIONS

CRC: Colorectal cancer
CTD: Comparative Toxicogenomics Database
EMT: Epithelial-mesenchymal transition
ESR2: Estrogen receptor beta
IGF: Insulin-like growth factor
OB: Oral bioavailability
QEDw: Quantitative estimate of the drug-likeness
T2D: Type 2 diabetes
